# Co-drug delivery of regorafenib and cisplatin with amphiphilic copolymer nanoparticles: enhanced *in vivo* antitumor cancer therapy in nursing care

**DOI:** 10.1080/10717544.2020.1815897

**Published:** 2020-09-16

**Authors:** Zhe Zhou

**Affiliations:** Department of Oncology, Huaihe Hospital of Henan University, Kaifeng, China

**Keywords:** Combinational delivery, cancer, apoptosis, in vivo antitumor efficacy

## Abstract

Cancers continue to be the second leading cause of death worldwide. Despite the development and improvement of surgery, chemotherapy and radiotherapy in cancer management, effective tumor ablation strategies are still in need due to high cancer patient mortality. Hence, we have established a new approach to achieve treatment-actuated modifications in a tumor microenvironment by using synergistic activity between two potential anticancer drugs. Dual drug delivery of Regorafenib (REGO) and Cisplatin (PT) exhibits a great anticancer potential, as REGO enhances the effect of PT treatment of human cells by providing stability of the microenvironment. However, encapsulation of REGO and PT fanatical by methoxypoly(ethylene glycol)-block-poly(D, L-lactic acid) (PEG-PLA in termed as NPs) is incompetent owing to unsuitability between the binary Free REGO and PT core and the polymeric system. Now, we display that PT can be prepared by hydrophobic coating of the dual drug centers with dioleoylphosphatidic acid (DOPA). The DOPA-covered PT can be co-encapsulated in PLGA NPs alongside REGO to stimulate excellent anticancer property. The occurrence of the PT suggestively enhanced the encapsulations of REGO into PLGA NPs (REGO-PT NPs). Further, the morphology of REGO NPs, PT NPs, and REGO-PT NPs and nanoparticle size was examined by transmission microscopy (TEM), respectively. Furthermore REGO-PT NPs induced significant apoptosis in human lung A549 and ovarian A2780 cancer cells by in vitro. The morphological observation and apoptosis were confirmed by the various biochemical assayes (AO-EB, Nuclear Staining and Annexin V-FITC). In a xenograft model of lung cancer, this nanotherapy shows a durable inhibition of tumor progression upon the administration of a tolerable dose. Our results suggest that a hydrophobic and highly toxic drug can be rationally converted into a pharmacologically efficient and self-deliverable nursing care of nanotherapy.

**Highlights**
- Dual drug delivery of Regorafenib (REGO) and Cisplatin (PT) exhibits a great anticancer potential, as REGO enhances the effect of PT treatment of human cells by providing stability of the microenvironment.
- REGO-PT NPs induced significant apoptosis in human lung A549 and ovarian A2780 cancer cells by in vitro.
- The morphological observation and apoptosis were confirmed by the various biochemical assayes.
- In a xenograft model of lung cancer, this nanotherapy shows a durable inhibition of tumor progression upon the administration of a tolerable dose.

## 1. Introduction

Nanoparticle-based drug delivery systems have been developed as a valuable system among other important methods for improved malignancy treatment. Appropriately structured nanoparticles can isolate the medications from the circulatory system and evade being eliminated by the renal system (Hu et al., 2017; Zhang & Tung, 2017; Wang et al., 2018; Hou et al., 2020; Wu et al., 2020). These nanoparticles have an advanced system to deliver anticancer medications to targeted locations and decrease nonspecific harm to the target tissues, brought about through enhanced permeability and retention (EPR) effects (Ge and Liu, 2013; Kumar et al., 2013; Florek et al., 2017). Moreover, nanoparticle frameworks offer stable watery scattering of medications by surface adjustment and shield medications from degradation, resulting in improved anticancer action (Ambrogio et al., 2013; Zhou et al., 2014; Shen et al., 2016; Wang et al., 2017; Yang et al., 2018; Dibaba et al., 2019; Chen et al., 2020).

Platinum metal complexes turn out a mainstay in cancer treatments and encompass some of the most powerful and progressive chemotherapeutic drugs (Esteban-Fernández et al., 2010; Cao et al., 2016; Li et al., 2017; Wlodarczyk et al., 2018). Despite surgical removal of tumors and various therapies scilicet radiation, chemo, immune, hormone, stem cell, precision medicine etc., shield people to stop dying from cancer, highly and inevitably used platinum chemotherapy saves 50–70% of all patients’ life howbeit with few drawbacks and side effects (AARON WOLD, 1971; Liu et al., 2016; Zhang et al., 2017; Wang et al., 2019). Mitigating efforts to vanquish the drawbacks triggered the exploration of more potent metallodrugs concerned with least toxicity by cancer cell selectivity, structural diversity, redox activity with amicable biochemical (bio-mimicking ability and ligand exchange kinetics) properties (Li et al., 2017; Zhang et al., 2017; Zhao et al., 2017; Lajous et al. 2018). At right time after some of the platinum compounds imprinted their promising in vitro anticancer and in vivo antitumor properties in the frontline of anticancer metallotherapeutics by addressing the aforementioned criterions with different modes of anticancer activities (Zhao et al., 2017).

As an oral multikinase inhibitor, Regorafenib (REGO) provides antiangiogenic activity in various tumor types by the inhibition of vascular endothelial growth factor receptors (VEGFR), tyrosine kinase with immunoglobulin and epidermal growth factor homology domain 2 (TIE-2), platelet-derived growth factor receptor-β (PDGFR-β), and fibroblast growth factor receptor (FGFR) (Khan et al., 2020; Wang et al., 2020). The activity is correlated with suppression of cell proliferation, and induction of apoptosis by the inhibition of oncogenic kinases (KIT, RET, RAF-1, BRAF and mutant BRAF). The US Food and Drug Administration (FDA) approved it for treating gastrointestinal stromal or metastatic colorectal cancer (mCRC) (Roser et al., 2018; Zhao et al., 2018; Weeramange et al., 2019). In 2017, FDA approved REGO for a second-line therapy in previously sorafenib treated HCC patients. Some commercial products of non-biodegradable microspheres have come into the market already such as DC Beads and Hepasphere™ for TACE. Drugs especially the positively charged were usually shielded by ion exchange approach, and the drug elution kinetics were determined by the ionic environment of physiological fluids (Liu et al., 2019).

Combination therapy can be performed via co-administration of a supplementary cancer drug along with a sensitizer. The interfaces within potential anticancer drugs rely on the dose ratios between the two medications and can be potentially incompatible (Li and Finley, 2018; Liu et al., 2019; Yang et al., 2019). Consequently, the importance of preserving a beneficial ratio to maintain a synergistic relationship between two drugs through nanoparticles (NPs) formulations cannot be ignored. The procedure of encapsulating several anticancer drugs in individual NPs has proved to be problematic because the drugs have to preserve their important physicochemical properties (Li & Finley, 2018; Liu et al., 2019; Yang et al., 2019). Hence, nanoformulations that are prepared by encapsulating numerous medications with varied physico-chemical belongings while preserving controlled ratios are preferred for drug delivery within the body tissues.

In this work, we have described a nanoplatform formed by encapsulation of two potential drugs into PLGA nanoparticles (REGO-PT NPs) via a nanoprecipitation method. Furthermore, in vitro cytotoxicity of the drug-loaded nanoparticles was examined in human lung (A549) and ovarian (A2780) cancer cells using an MTT assay. Additionally, we examined morphological changes in the treated cells by dual staining (AO-EB) and nuclear staining methods. Apoptosis were confirmed by the flowcytometry analysis. To establish the potential of this REGO-PT NPs fabrication to be translated to the clinic, we evaluated the antitumor efficacy in mouse models of human A549 tumor xenografts. Our nanoparticle-mediated delivery platforms provide a simple, broadly applicable strategy to effectively enhance the potency and safety of molecularly targeted agents that have previously been limited to tumor administration.

## 2. Materials and methods

### 2.1. Materials

PT and REGO were purchased from TCI (Shanghai, China). Hydrolyzed Polyvinyl alcohol (PVA, 85–90%, Mol. Wt of 30 K–50K Da) were obtained from TCI, China. PLGA polymers (monomer ratio 50:50; MW 7 K Da) was acquired from J&K, China.

### 2.2. Methods

#### 2.2.1. Encapsulation of REGO and PT in REGO-PT NPs

An oil/water solvent evaporation technique adapted to encapsulation of PT and REGO in PLGA-NPs (Zhao et al., 2014; Shao et al., 2017; Govindasamy et al., 2019). Briefly, DOPA-coated PT (50 µg) cores and REGO (50 µg) were added to a PLGA-NP solution in CHCl_3_ (100 mg in 350 µl). The emulsified 9% PVA was mixed into chloroformic solution in 3 mL PBS solutions. The emulsions were stirred for 24 h, and evaporate the organic solvents. PT- and REGO-loaded PLGA nanoparticles (REGO-PT NPs) were kept at −20 °C to be used for future studies.

A water/oil/water double emulsion solvent evaporations technique were used to fabricate the PLGA-NPs containing DOPA-coated PT, REGO. Briefly, TMR-dextran (200 µl) was blended into a PT and REGO polymeric solutions in CHCl_3_ with sonication’s. These emulsions were consequently blended in a PVA-PBS solutions, left for solvents evaporations. The emulsions were stirred for 24 h, and evaporate the organic solvents.

### 2.3. Examination of in vitro drug release

Assessment of in vitro drug release kinetics was performed using a dialysis diffusion technique. REGO-PT NPs (3 ml), and PT and REGO (0.1 mg/ml equivalent concentration) solutions were placed into the end-wrapped dialysis covers. Next, they were retained into 20 ml of discharging medium comprising 0.2% Tween-80 in PBS pH 7.4. By stirring at 100 rpm on a detour shakers at 37 °C, the drug release medium was removed and an equivalent size of new medium was added. The drug-releasing profiles of PT and REGO were examined using an UV − vis spectrometer (Boyd et al., 2019; Ibrahim et al., 2020; Li et al., 2020).

### 2.4. In vitro cytotoxicity

A549 and A2780 cells were obtained from the Cell Bank of Beijing. The cells were maintained in RPMI 1640 culture and Dulbecco’s modified Eagle’s (DMEM) medium supplemented with 10% fetal bovine serum (FBS) and 100 ml^−1^ penicillin. Then, A549, and A2780 cells were incubated in a humid atmosphere with 5% CO_2_ at 37 °C. In vitro biochemical staining was obtained from Cell Signaling (China).

### 2.5. Mtt assay

A549 and A2780 cells were cultured in 96-well plates (4000 cells per well) and incubated for 24 h at 37 °C. Free PT, Free REGO, PT NPs, REGO NPs, and REGO-PT NPs were well dissolved in DMSO and the final contents of DMSO were less than 0.2% (v/v) to avoid the solvent impacting cell viability. Then the cells were treated with various concentrations of Free PT, Free REGO, PT NPs, REGO NPs, and REGO-PT NPs for 24 h. Experiments were performed in triplicate and the medium without the samples were served as the control. After 24 h, 30 μL of 3-[4,5-dimethylthiazol-2-yl]-3,5-diphenyl tetrazolium bromide (MTT) in phosphate buffered saline solution at a concentration of 5 mg mL − 1 was added into each well and incubated at 37 °C for 5 h. Then the medium with MTT was removed and 100 μL of DMSO was added to dissolve the formazan crystals formed. The absorbance of each sample at 492 nm on a microplate reader (Multiskan FC, Thermo Scientific). Cell viability was calculated as follows: cell viability (%) = [absorbance of each well/absorbance of control well] × 100. Graph was plotted between % of cell inhibition and concentration of the test samples. From this plot, the IC_50_ value was calculated.

### 2.6. Apoptotic staining

The morphological changes of the A549 cells were examined by biochemical staining, including acridine orange-ethidium bromide (AO-EB) and Hoechst 33344 staining. After incubating for 24 h, the cells were seeded at a concentration of 1 × 10^4^ onto 48-well plates. The cells were treated with Free PT, Free REGO, PT NPs, REGO NPs, and REGO-PT NPs at 2.5 µM concentration for 24 h. On the following day, the staining solution was added. After incubating the plates with the staining solution, the plates were washed with PBS three times (Mohamed Subarkhan et al., 2016; Mohamed Kasim et al., 2018; Balaji et al., 2020). Images were obtained using a fluorescence microscope (Accu Scope EXI-310) at a magnification of 20×.

### 2.7. Flow cytometry/annexin V-PI staining

The flow cytometry examination was examined by using the Apoptosis Detection Kit of fluoresceinisothiocyanate (FITC) (Cell Signaling, China) utilized to confirm the apoptotic ratio of A549 cells. The cells were treated with Free PT, Free REGO, PT NPs, REGO NPs, and REGO-PT NPs at 2.5 µM concentrations for 24 h. The cells were washed thrice by using trypsin, and suspended in 1 × binding buffer (500 μL) with FITC Annexin V (5 μL) and of PI (10 μL). After 20 min incubation, the samples were analyzed by flow cytometry. The obtained results were investigated with the BD FACS CantoTM II flow cytometer.

### 2.8. Evaluation of the in vivo drug toxicity

The in vivo drug toxicity was investigated in ICR mice (4–5 weeks old). Healthy ICR mice were randomly divided into 5 groups (*n* = 10 mice per group). Drugs were injected through the tail vein on days 0, 3, and 6. Mice were injected with Free PT (2.5, and 5 mg/kg, Ciaplatin equivalent dose), Free REGO (2.5, and 5 mg/kg), PT NPs (2.5, and 5 mg/kg), REGO NPs (2.5, and 5 mg/kg), and PT-REGO NPs (2.5, 5, and 10 mg/kg). Saline were injected as a control. The body weights of the mice were recorded every three days (Zhou et al., 2016; Ghosh et al., 2018; Salie et al., 2019).

### 2.9. Histologic analysis

For histological analysis, the organs from the sacrificed mice were excised at the end of the treatments with various drugs. After being fixed in 4% formaldehyde and embedded in paraffin, the tumor tissues and organs were further sectioned into 5 μm slices for hematoxylin and eosin (H&E, Sigma) staining. The H&E-stained tissues were imaged by fluorescence microscopy (Olympus, IX71).

### 2.10. In vivo antitumor activity

BALB/c nude mice (4–5 weeks old) were used for the evaluation of the antitumor activities of the nanotherapies. The human prostate cancer cell line A549 was grown to 80% confluence in 90 mm tissue culture dishes. After cell harvesting, the cells were resuspended in PBS at 4 °C to reach a final concentration of 2.5 × 10^7^ cells/mL. The right flanks of the BALB/c nude mice were subcutaneously injected with 200 μL of a cell suspension containing 5 × 10^6^ cells. At 14 days after implantation, the tumors reached approximately 60 mm^3^ in volume, and then the animals were randomly divided into five groups (*n* = 7 mice per group). Mice bearing A549 tumor xenografts were injected intravenously with samples solutions (Free PT at 2.5 mg/kg, Free REGO at 2.5 mg/kg, PT NPs at 5 mg/kg, REGO NPs at 5 mg/kg, and PT-REGO NPs at 10 mg/kg) three times on days 0, 3, and 6. Saline were also injected as a control. Tumor volumes and body weights were monitored and recorded for 33 days. The lengths (L) and widths (W) of the tumors were measured with calipers, and the tumor volume was calculated by the following formula: V = (L × W_2_)/2, where W is shorter than L. Mice were sacrificed by CO_2_ inhalation at the endpoint of the study.

### 2.11. Data analysis

The data analysis of different groups was conducted with one-way ANOVA in GraphPad Prism 5 software. The significant level were considered at *p* < .05 and greatly significant at *p* < .001. All data are presented as mean ± SD. (Unless otherwise stated, *n* = 3).

## 3. Results and discussion

### 3.1. Structural morphology and characterization

**Figure 1.**
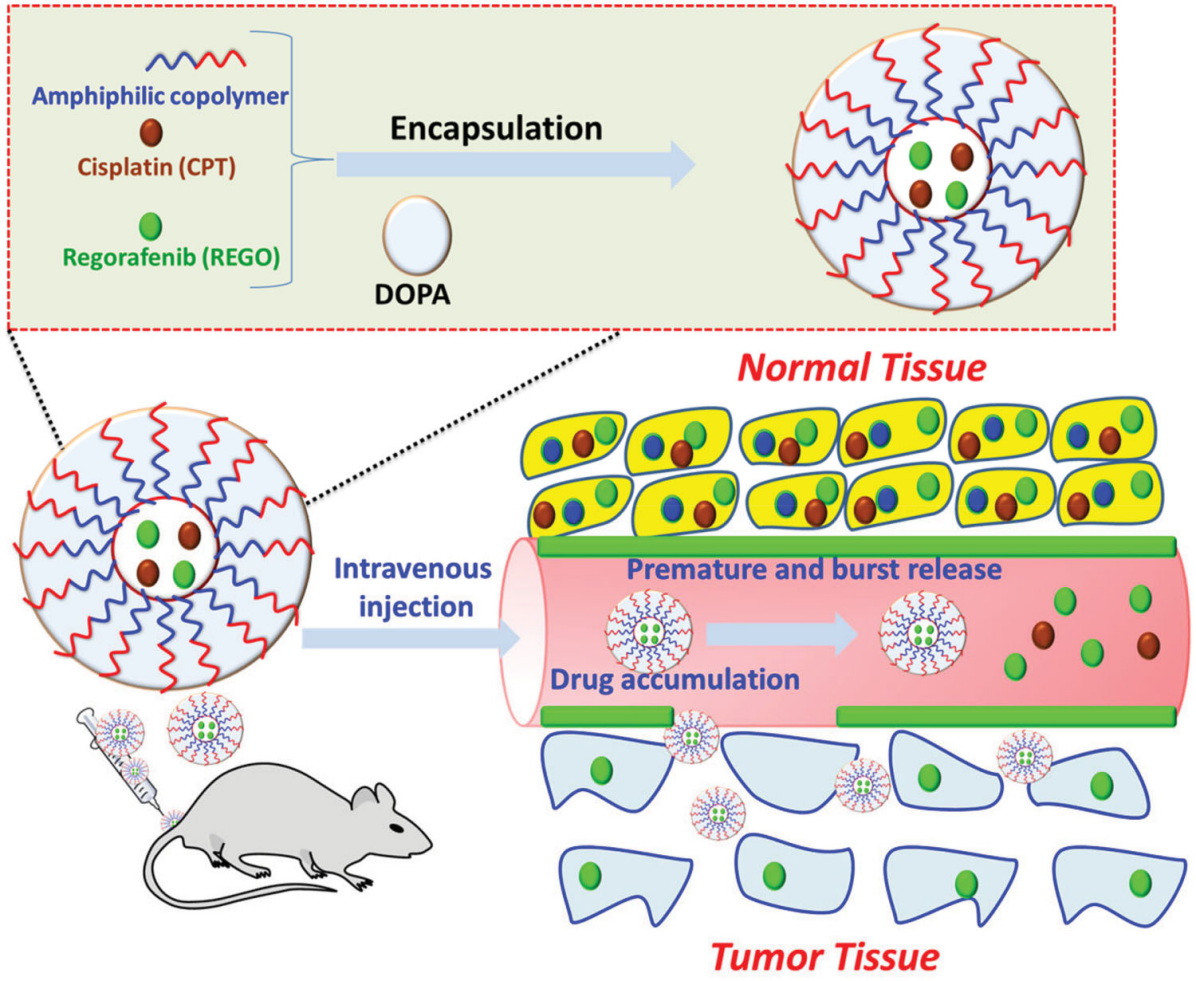
A graphic representation of the encapsulation of REGO and PT into amphiphilic polymers to form REGO-PT NPs for the treatment of cancer therapy.

Our achievement in proficiently stacking of Cisplatin (PT) and Regorafenib(REGO) into PLGA-NPs (designated as REGO-PT NPs) proposals another chance to co-deliver two medications for blend treatment. For instance, hydrophobic PT and REGO can be built into REGO-PT NPs simultaneously with other hydrophobic antitumor medications, such as REGO and paclitaxel. REGO was preferred for this study and its centers were embodied into REGO-PT NPs close to PT, because of its cooperative energy with PT. The main procedure of stacking of REGO and PT inside REGO-PT NPs is shown in Figure 1. REGO and PT are incorporated in the polymer framework of REGO-PT NPs done by hydrophobic interaction. Hence, the insertions are restricted by similarities concerning REGO and PT and their hydrophobic interaction with the co-polymer. Self-assembled nanoparticles (REGO-PT NPs) were formed spontaneously with 4 mg/ml PT and 8 mg/ml REGO by employing intermolecular hydrophobic interactions between the lipophilic core of REGO and PT, as depicted in Figure 1.

**Figure 2.**
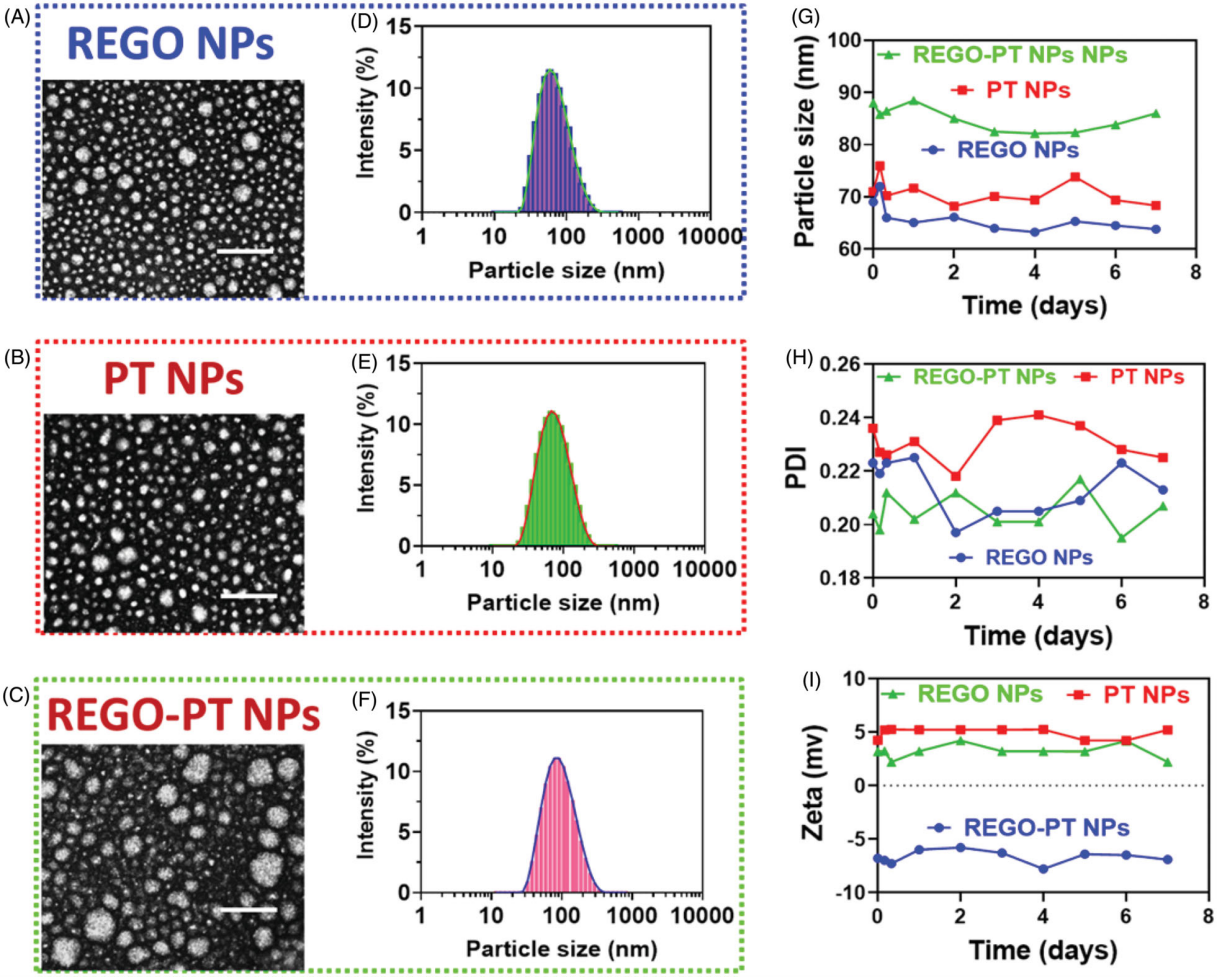
Characterization of the nanoparticles. (A–F) Morphology and particle size of REGO NPs, PT NPs and REGO-PT NPs under a transmission electron microscope after negative staining with sodium phosphotungstate solution (2%, w/v). Scale bar: 20 nm. Particle size distribution of REGO NPs, PT NPs and REGO-PT NPs analyzed by dynamic light scattering via a Zetasizer. (G-I) Stability of the REGO NPs, PT NPs and REGO-PT examined by the dynamic light scattering.

The effects of the morphological surface of the hydrothermally prepared REGO NPs, PT NPs, and REGO-PT NPs were investigated through TEM analysis. The results as shown in Figure 2(A–C) depicts the creation of REGO-PT NPs. Additionally, morphological changes the synthesized polymeric nanoparticles were analyzed by HR-TEM. The nanocomposite was composed of from agglomerated clusters of well-shaped hydroxyapatite nanocomposites (Figure 2(A–C)). The size of the REGO-PT NPs was examined by dynamic light scattering (DLS) analysis. The diameters of REGO NPs, PT NPs, and REGO-PT NPs measured from TEM images were in the range of 63.8 ± 2.3, 69.3 ± 1.8, and 83.2 ± 1.9 nm (Figure 2(D–F)) and the Polyplexes index were 0.277 ± 0.05, 0.252 ± 0.05, and 0.159 ± 0.02 for REGO NPs, PT NPs, and REGO-PT NPs respectively, which is in agreement with the results of light scattering measurements and gives clear evidence of the size of the nanoparticles compared to those analyses by TEM (Figure 2(D–F)). The stability of the REGO NPs, PT NPs, and REGO-PT NPs in PBS media was examined by determining the particle size of the REGO NPs, PT NPs, and REGO-PT NPs by dynamic light scattering. Polyplexes index, specifically REGO NPs, PT NPs, and REGO-PT NPs, at an NPs ratio of 100:1 were organized and incubated for 30 min at 37 °C in order to confirm complete polyplex formation (Figure 2(G–H)). All the experiments were repeated three times. Additionally, the zeta potential and the stability of REGO NPs, PT NPs, and REGO-PT was determined to be 5.2 ± 0.4, 6.8 ± 0.5 and −6.3 ± 0.3 mV (Figure 2(I)) by DLS. Hence, this fabrication approach for REGO-PT NPs produced favorable particle sizes, which may potentially increase intratumoral accumulation. In addition, the values of EE and the percentages of DL was determined by HPLC analysis. As a result, the EE values were 91.0 ± 0.8% for REGO-PT NPs, respectively. The percentage of DL were 4.3% for REGO-PT NPs, respectively.

### 3.2. Controlled release of REGO-PT NPs

**Figure 3.**
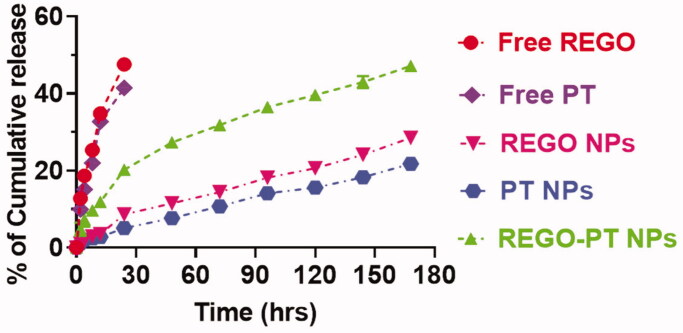
Drug release profiles (REGO and PT) from the REGO NPs, PT NPs, and REGO-PT NPs against PBS containing 0.3% polysorbate 80.

Controlled release of REGO-PT NPs plays a vital role in the size, solubility, degradation, and drug loading by the nanoparticle frameworks. It is predictable results to confirm the drug release profile shows the PT + REGO-loaded REGO-PT NPs reserve an enhanced efficiency to the frameworks. In contrast, if the drugs not deceived, a reckless and undesired untimely discharge will occurs. These methods provide clues to the production of shell holes that permit the discharge of drugs. The controlled drug release was measured via physical and chemical analysis of the REGO-PT NPs and the encapsulation properties of the drugs. These dialysis methods were utilized to examine the outcomes of controlled release of the drugs encapsulated in the REGO-PT NPs and those associated with the Free PT and REGO. The controlled release experiment was conducted in PBS at a pH of 7.2 at 37 °C. The controlled release profiles of the combination of PT and REGO loaded in the REGO-PT NPs displayed an initial release in about 5 h monitored via sluggish release for six days (Figure 3). First 10 h, half of the PT and REGO was discharged after the REGO-PT NPs formations. Subsequently, later 24 h, a gentle release of 40% to 50% was observed. These results indicate that the conjugation of PT and REGO on the surface of the PLGA-NPs (REGO-PT NPs) did not show any adverse effect on the controlled release by these nanocomposites.

### 3.3. *In vitro* cytotoxicity

**Figure 4.**
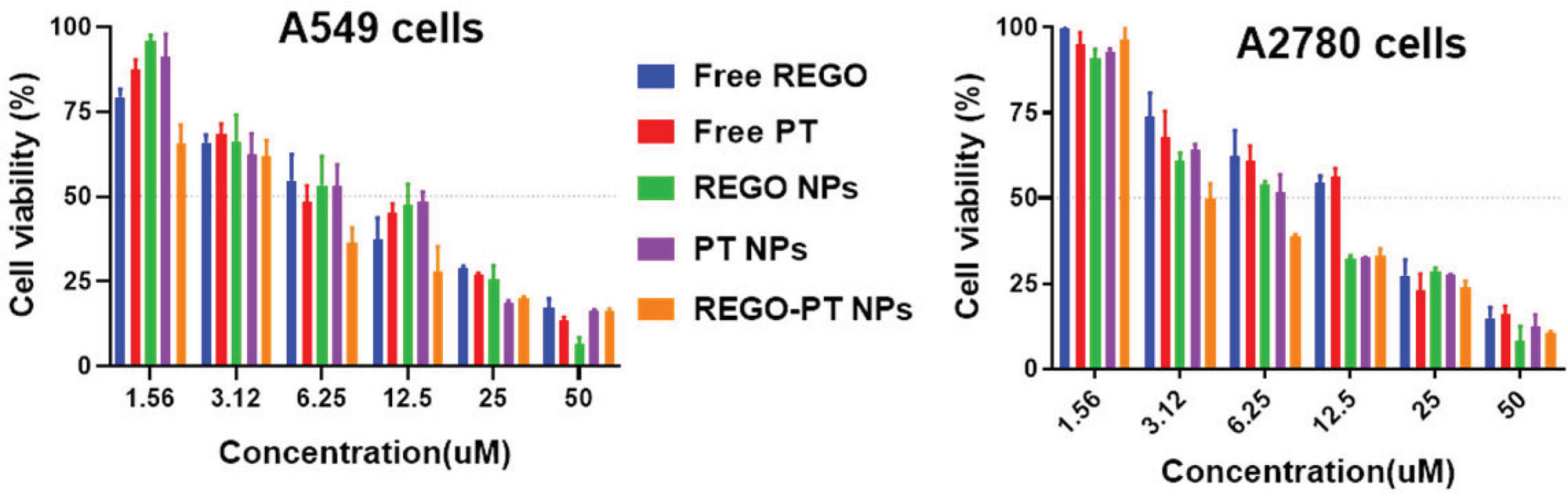
In vitro cytotoxicity of Free PT, Free REGO, PT NPs, REGO NPs, and REGO-PT NPs were evaluated in A549 and A2780 cancer cells. Cell viability was examined by the MTT assay after 24 h of drug incubation.

After successful fabrications of REGO-PT NPs, we performed an MTT assay to evaluate the cytotoxic effects of Free PT, Free REGO, PT NPs, REGO NPs, and REGO-PT NPs on cancer cell lines, comprising A549 and A2780 cancer cells. Following treatments with the drugs for 24 h, the cells viability was monitored, and minimum-inhibitory concentrations (IC_50_) were obtained from the dose-dependent curve (Figure 4). Surprisingly, REGO-PT NPs displayed substantial improvement in cytotoxicity of the cancer cells. For instance, in A549 cell lines, IC_50_ of 10.91 ± 11.12, 10.35 ± 1.22, 9.05 ± 2.11, 9.46 ± 0.98 and 6.62 ± 0.97 were observed for free PT, free REGO, PT NPs, REGO NPs, and REGO-PT NPs, respectively. In A2780 cell lines, IC_50_ of 19.27 ± 3.30, 17.70 ± 2.54, 11.20 ± 0.98, 10.22 ± 1.87, and 7.16 ± 2.80 for Free PT, Free REGO, PT NPs, REGO NPs, and REGO-PT NPs were observed, respectively. The enhanced cytotoxicity of the REGO-PT NPs was owing to the entire release of the double potential anticancer medications into the tumor cells. The hydrophilic molecules of PLGA dispense the aqueous layer via a lipid bilayer for cell membrane penetration. Thus, the enhancement of cellular uptake requires the cell membrane nucleosides delivery for the proteins.

### 3.4. Morphological changes in A549 cancer cells

**Figure 5.**
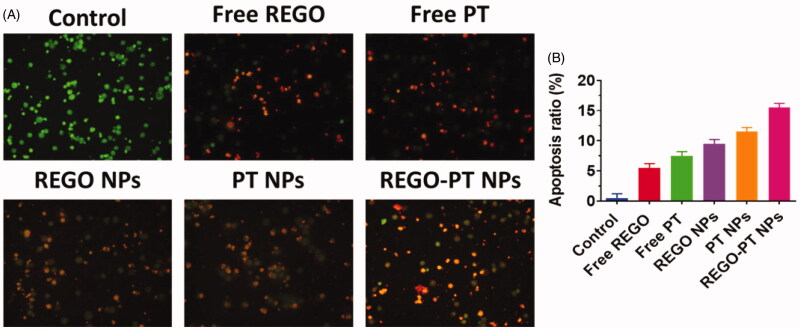
Dual AO/EB staining assay for examining Free PT, Free REGO, PT NPs, REGO NPs, and REGO-PT NPs-induced cell death in A549 cells. The cells were treated with Free PT, Free REGO, PT NPs, REGO NPs, and REGO-PT NPs at 2.5 µM concentration for 24 h. B) Quantification of apoptosis ratio. The cells were quantified by image J software.

Dual staining AO-EB is a qualitative technique used to identify live, early, late apoptotic, and necrotic cancer cells using fluorescent images to observe morphological changes in the nucleus of cells. AO permeates the intacts membranes of usual and early apoptotic cell and binds to DNA, which fluoresces uniform green in normal cells and as patches in early apoptotic cells due to chromatin condensations. In difference, EB is only penetrable in the incapacitated membrane of late apoptotics and necrotics cell, where it fluoresces as bright orange patch through its bindings to DNA fragment or apoptotic bodies in late apoptotic cells, and as a unchanging orange fluorescence in the necrotic cell, due to have the nuclear changes in the morphology of viable cell. AO-EB-stained A549 cells were incubated with Free PT, Free REGO, PT NPs, REGO NPs, and REGO-PT NPs for 24 h. As presented in Figure 5(A), the presence of orange with reddish fluorescence with chromatin fragmentation after treatment of A549 cells treated with Free PT, Free REGO, PT NPs, REGO NPs, and REGO-PT NPs suggested that the REGO-PT NPs largely induced apoptosis in A549 cells (Figure 5(B)).

**Figure 6.**
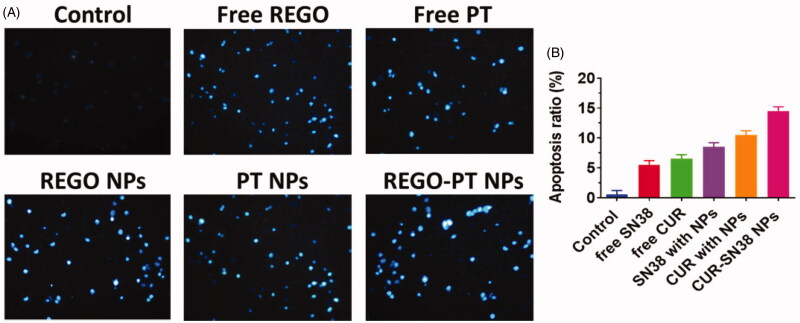
Nuclear (Hoechst 33258) staining assay for examining Free PT, Free REGO, PT NPs, REGO NPs, and REGO-PT NPs-induced cell death in A549 cells. The cells were treated with Free PT, Free REGO, PT NPs, REGO NPs, and REGO-PT NPs at 2.5 µM concentration for 24 h. B) Quantification of apoptosis ratio. The cells were quantified by image J software.

Hoechst 33258 staining was used to observe chromatin fragmentation, bi- and/or multinucleation, cytoplasmic vacuolation, nuclear swelling, cytoplasmic bleating, and late apoptosis in cancer cells by visualizing dot-like chromatin condensation. Hoechst-33258–stained A549 cells were incubated with Free PT, Free REGO, PT NPs, REGO NPs, and REGO-PT NPs for 24 h. As displayed in Figure 6(A), the presence of blue fluorescence with chromatin condensation after treatment of A549 cells treated with Free PT, Free REGO, PT NPs, and REGO NPs suggested that the REGO-PT NPs largely induced apoptosis in A549 lung cancer cells (Figure 6(B)).

### 3.5. Apoptosis in A549 cancer cells

**Figure 7.**
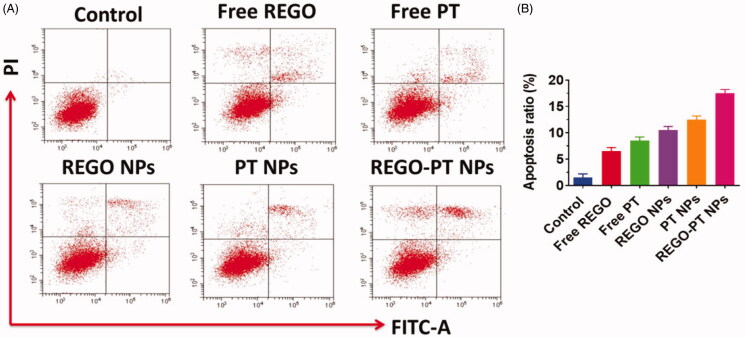
A) Apoptotic analysis of A549 cells using flow cytometry. The cells were treated with Free PT, Free REGO, PT NPs, REGO NPs, and REGO-PT NPs at 2.5 µM concentration for 24 h and then stained with FITC annexin V/PI for flow cytometry analysis. B) Apoptosis ratio of A549 cells.

Apoptosis may be reckoned as an important obstacle for a damaged cell to become malignant tumors. Since the complexes promote apoptosis induction in cancer cells, flow cytometry using annexin V-FITC/propidium iodide (PI) double staining was carried out for the quantitative discrimination of apoptotic cells. Phosphatidylserine (PS) is a cell cycle signaling phospholipid located inner side of the membrane of a healthy cell but is reverted to the outer membrane for recognition by neighboring cells at the time of apoptosis. Hence, the translocation of phosphatidylserine is a morphological hallmark of apoptosis and can be spotted by its binding with fluorescently labeled annexin V which in turn detected by flow cytometry. Further the addition of PI to annexin V stained cells is used to discriminate and concomitantly quantify the live cells (lower left quadrant-annexin V(-)/PI(-)), early apoptotic cells (upper left quadrant-annexin V(+)/PI(-)) and late apoptotic cells (upper right-quadrant-annexin V(+)/PI(+)) using FACS. As projected in Figure 7(A), the incubation of Free PT, Free REGO, PT NPs, REGO NPs, and REGO-PT NPs with A549 cells conspicuously induced apoptosis. It is worth to note that the titled complexes induce apoptosis even at very low concentrations which is less than their IC_50_. In comparison with control, the cell population was higher (6-9%) in annexin V(+)/PI(-) (upper left) quadrant indicating the induction of early apoptosis (Figure 7(B)). This effect was ascertained to be high for REGO-PT NPs than the Free PT, Free REGO, PT NPs, REGO NPs analogous with the results of MTT, and AO-EB staining assays. It is to note that the test samples displayed comparatively better apoptotic induction on A549 cells.

### 3.6. Histological evaluation for systemic toxicity

**Figure 8.**
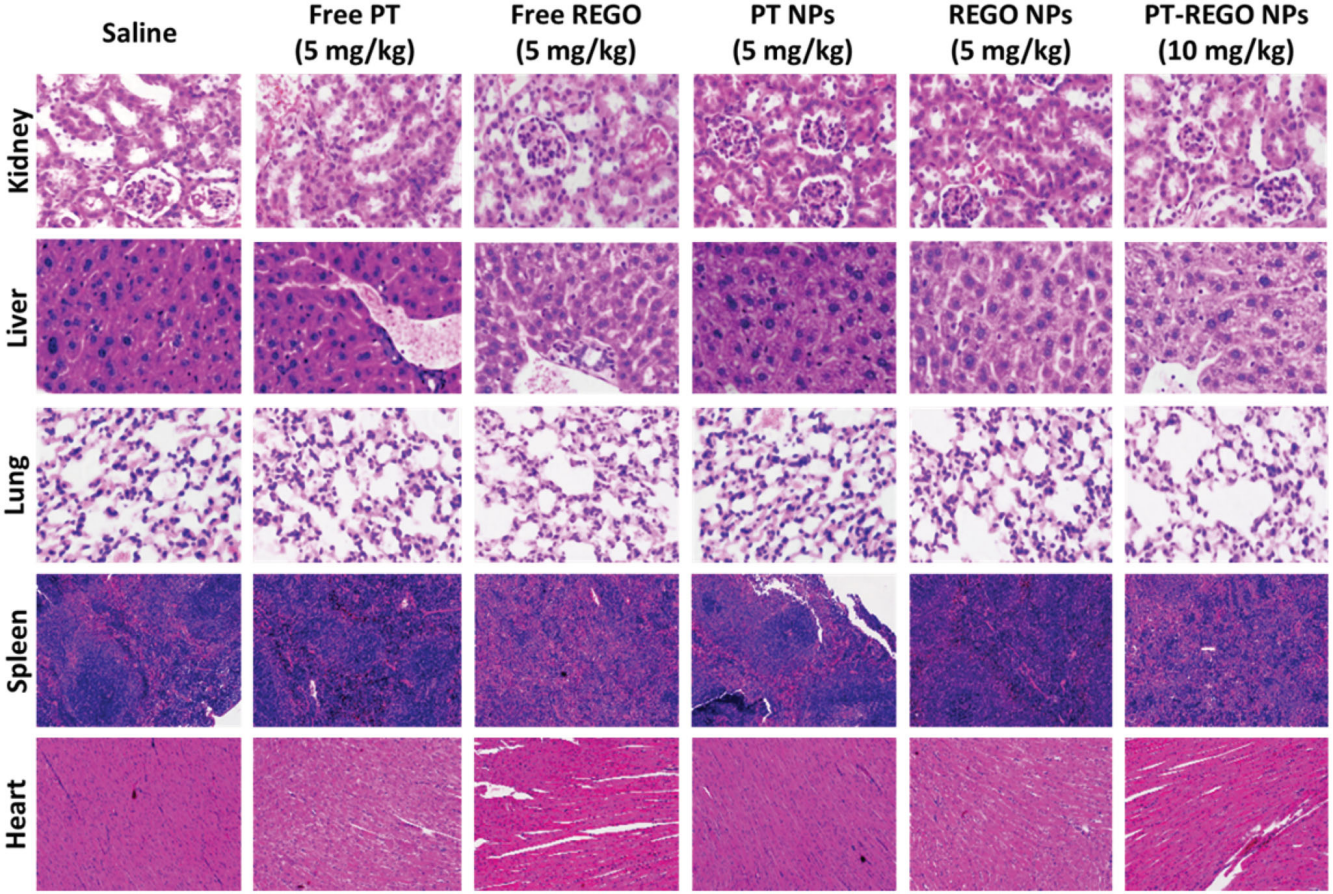
H&E staining of the major organs (kidney, liver, lung, spleen and heart) excised from different treatment mice groups. Scale bar: 100 μm.

The efficiency of anticancer chemotherapeutic drugs is mainly validated by its selective action toward cancer tissues leaving the normal organs undamaged. After the verification of low systemic toxicity in the mice injected with Free PT (2.5, and 5 mg/kg), Free REGO (2.5, and 5 mg/kg), PT NPs (2.5, and 5 mg/kg), REGO NPs (2.5, and 5 mg/kg), and PT-REGO NPs (2.5, 5, and 10 mg/kg), histological analyses were carried out to identify the structural changes in the tissues of vital of organs inclusive of heart, liver, spleen, lung, and kidney of the mice treated with Free PT, Free REGO, PT NPs, REGO NPs, and PT-REGO NPs and compared with control, the saline received mice. Figure 8 represented the histological sections of the heart, liver, spleen, lung, and kidney stained with hematoxylin and eosin (H&E).The photomicrographs of the liver and spleen of the control, Free PT, Free REGO, PT NPs, REGO NPs, and PT-REGO NPs treated groups displayed normal cellular morphology. Under optical microscopy examination, the heart, lung, and kidney of Free PT, Free REGO, PT NPs, REGO NPs, and PT-REGO NPs treated animals showed normal cardiac muscle fibers, normal alveolar, and normal glomerular histological characteristics respectively which were found to be similar histological architecture as those of the control group with no treatment-related inflammatory response.

### 3.7. In vivo antitumor efficacy in A549 xenograft tumor model

**Figure 9.**
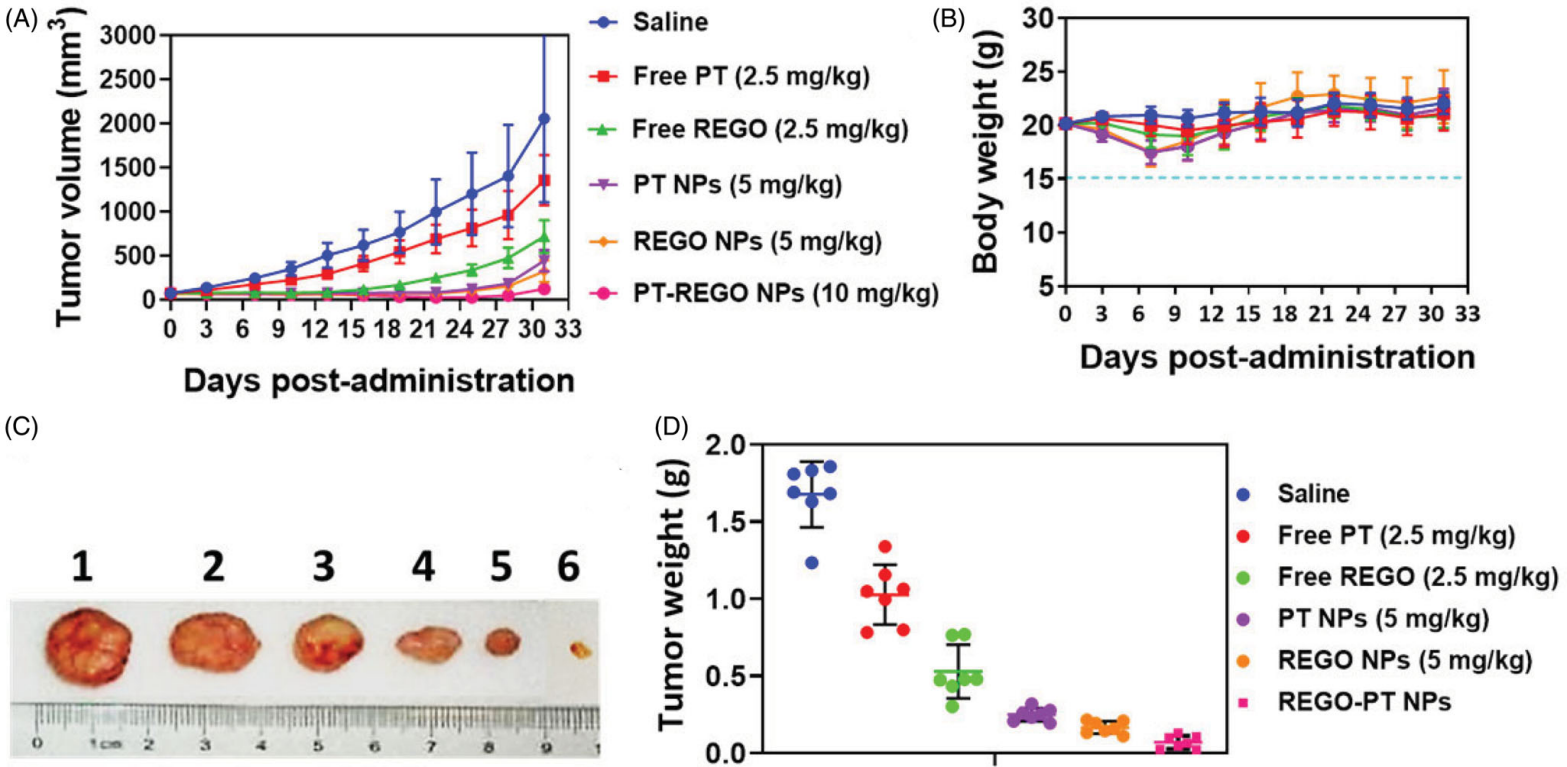
In vivo antitumor activity of Free PT, Free REGO, PT NPs, REGO NPs, and PT-REGO NPs compared to saline. A549 tumor xenograft-bearing Balb/c nude mice were administered with various drugs via intravenous injection at days 0, 3 and 6. A) Changes in tumor volumes. B) Body weights. C) Represent tumor photograph. D) Tumor weights. The data are presented as the means ± SD (*n* = 7).

Considering the promising in vitro biological activity profiles, the in vivo pharmacological efficacy was further investigated in a A549 xenograft tumor model. In the experimental process, body weight of animals in each group was stable. It suggested that the experimental doses in all groups were tolerable. As shown in Figure 9(A–C), we found an obvious retardation of tumor growth for animals treated with Free PT, Free REGO, PT NPs, REGO NPs, and PT-REGO NPs, as compared to the control group. Specifically, nanoparticles delivering PT-REGO NPs more efficiently suppressed tumor growth than administered Free PT, Free REGO (Figure 9) panels a tumor site(s) via the EPR effect. Moreover, these PT-REGO NPs did not significantly affect the body weights of mice, indicating that the delivery materials and Free REGO have low systemic toxicity. Most importantly, treatment with the combination of PT-REGO NPs could significantly enhance the efficacy of chemotherapy for PT-REGO NPs, as evidenced by more remarkable slow-down for tumor growth in relative to the Free PT, Free REGO, PT NPs and REGO NPs group (*p* < .05). On day 33, animals in saline groups performed a high average tumor weight of 1.58 g (Figure 9(D)). The animals treated with Free PT, Free REGO, PT NPs, REGO NPs, and PT-REGO NPs exhibited lower mean tumor weight of 0.92 g, 0.50 g, 0.36, 0.29 and 0.09 g, respectively. A significantly lower mean tumor weight was obvious for PT-REGO NPs compared to Free PT, Free REGO (*p* < .05).

## 4. Conclusion

We developed REGO-PT NPs by encapsulating REGO and PT core to change the tumor microenvironment for improved drug accretion and additional anticancer activities. At first, PT was incorporated into REGO-PT NPs with effectual loading and encapsulation by direct self-assembly method. In this study, we showed that PT could be made hydrophobic by using an oil/water solvent evaporation method for drug delivery. These DOPA-covered PT centers were compatible with PLGA and could be co-encapsulated in REGO-PT NPs. The closeness of the PT centers fundamentally developed the epitome of REGO into PLGA-NPs. The formation of the nanocomposite was confirmed by TEM electroscopic techniques displayed the crystallized structure of the nanocomposite. REGO-PT NPs comprising double PT and REGO led to remarkable apoptosis in human lung (A549) and ovarian (A2780) cancer cells. Further, morphological changes in the cells were monitored using dual staining and nuclear staining methods. AO-EB fluorescent staining and flow cytometry analysis reveal that the samples induce cancer cell death by apoptosis mechanism. Moreover, in vivo investigation in an A549 xenograft tumor model demonstrated the outstanding antitumor efficacy of REGO-PT NPs significantly in superior to the rest of the samples. In summary, the results of this study demonstrated that REGO-PT NPs for local delivery as a novel combination strategy may enhance the therapeutic potency for the treatment and nursing care of lung cancer, and has promising clinical implications in future.
